# Escalating Research Output After the USMLE Step 1 Pass/Fail Transition: Implications for Equity, Ethics, and the Orthopaedic Residency Match

**DOI:** 10.1177/23821205261450081

**Published:** 2026-06-11

**Authors:** Yzen Al-Marrawi, James R. Burmeister, Majd Al-Marrawi, Amir Al-Marrawi, Ismail Zazay, Aws Hammad

**Affiliations:** 1 Department of Foundational Medical Studies, 159878 Oakland University William Beaumont School of Medicine, Auburn Hills, USA; 2 2647 The Ohio State University, College of Arts and Sciences, Columbus, USA; 3 Department of Surgery, 12338 The University of Texas Medical Branch at Galveston, Galveston, USA; 4 Department of Orthopaedic Surgery, 7005 Corewell Health William Beaumont University Hospital, Royal Oak, USA

**Keywords:** orthopaedic surgery, residency match, research output, USMLE step 1, medical education, equity, graduate medical education, applicant competitiveness

## Abstract

**Background:**

Orthopaedic surgery remains one of the most competitive residency specialties in the United States. Following the 2022 transition of USMLE Step 1 to pass/fail, research output (RO) has emerged as a prominent screening metric for residency selection. However, longitudinal trends in RO and the impact of this policy change on applicant behavior have not been fully characterized.

**Methods:**

Aggregate data from the National Resident Matching Program (NRMP) Charting Outcomes in the Match reports from 2014 to 2024 were analyzed for U.S. allopathic applicants to orthopaedic surgery. RO was defined as the self-reported number of peer-reviewed publications, abstracts, and poster presentations. Descriptive analyses were performed, and linear trend modeling compared RO growth before the Step 1 policy change (2014–2020) and after implementation (2022–2024).

**Results:**

Average RO among matched applicants increased by 255% over the study period, rising from 6.7 in 2014 to 23.8 in 2024. Unmatched applicants demonstrated similar upward trends but consistently reported lower RO. From 2014 to 2020, RO increased modestly at approximately 1.3 research products per year. Following the Step 1 pass/fail transition, RO growth accelerated sharply to approximately 5.2 products per year between 2022 and 2024. During this period, matched applicants experienced a 44.24% increase in RO and reported 32% higher RO than unmatched applicants by 2024.

**Conclusions:**

Research output among orthopaedic residency applicants has risen dramatically over the past decade, with a pronounced acceleration following the Step 1 transition to pass/fail. These findings suggest a shift toward research quantity as a dominant selection metric, raising concerns regarding sustainability, equity, burnout, and ethical authorship practices. Ongoing ERAS reforms and adoption of holistic review frameworks may be necessary to ensure fair, meaningful, and transparent residency selection in orthopaedic surgery.

## Introduction

Orthopaedic Surgery remains one of the most competitive residency specialties in the United States, with a 2025 match rate of 69% for U.S. allopathic seniors.^1^ As the applicant pool grows, research output (RO), defined by the National Resident Matching Program (NRMP) as the cumulative number of peer-reviewed publications, abstracts, and presentations, has become an increasingly prominent screening metric.^2^ The 2022 transition of the United States Medical Licensing Examination (USMLE) Step 1 to pass/fail eliminated the primary numerical measure historically used to stratify applicants.^3^ Although intended to promote holistic review, this change has contributed to an intensified emphasis on alternative metrics, especially RO and Step 2 CK scores.^4^ Early evidence suggests that applicants may be altering their behavior in response to these shifting expectations, raising concerns about equity, sustainability, and ethical authorship practices in orthopaedic residency recruitment.

## Methods

Data were obtained from NRMP *Charting Outcomes in the Match* reports from 2014 to 2024 for U.S. allopathic applicants to Orthopaedic Surgery programs. RO was defined as the self-reported number of publications, abstracts, and poster presentations listed by applicants; these values were not independently validated. Data were compiled in Microsoft Excel and analyzed descriptively. Linear trend modeling was conducted in R to compare growth in RO before the Step 1 policy change (2014–2020) and after its implementation (2022–2024). Because the NRMP data are aggregate and observational, causal inference and adjustment for confounding variables were not possible.

## Results

**Figure 1.**
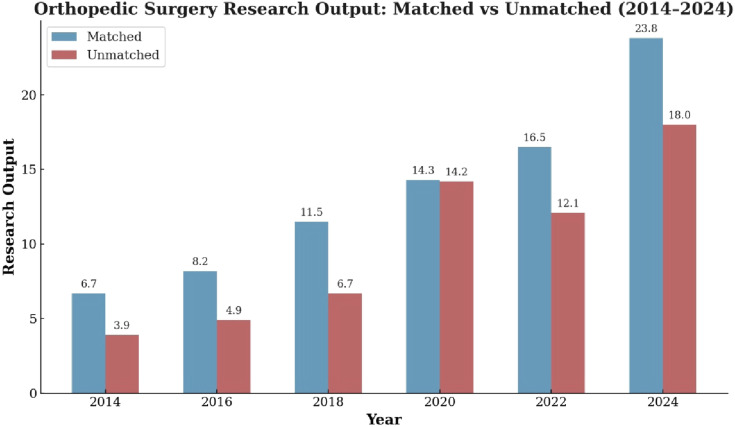
Average research output among matched and unmatched U.S. allopathic orthopaedic applicants, 2014–2024

**Figure 2.**
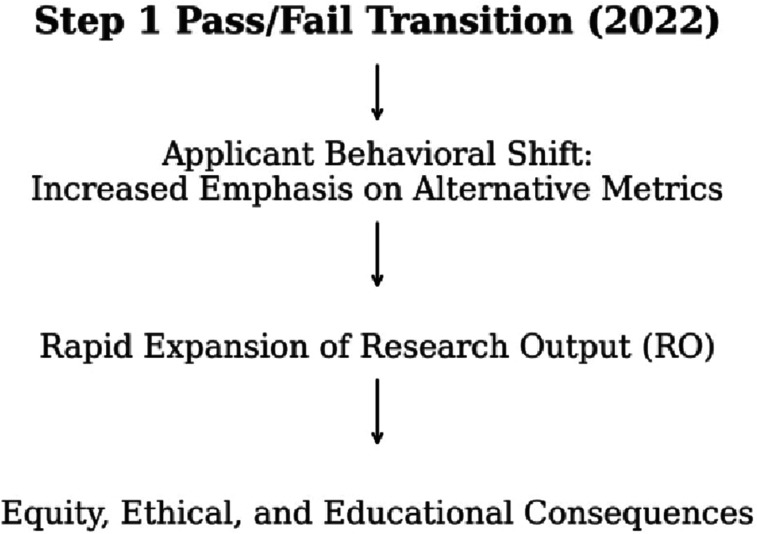
Conceptual model illustrating how the Step 1 pass/fail transition may influence applicant behavior, research output inflation, and downstream equity and ethical considerations

There was a substantial increase in RO among matched applicants over the past decade. The average RO rose by 255%, from 6.7 in 2014 to 23.8 in 2024. Unmatched applicants demonstrated similar upward patterns, although they consistently reported lower RO than matched applicants. Trend analysis revealed a modest, steady increase in RO from 2014 through 2020, with an approximate growth rate of 1.3 research products per year. Following the Step 1 transition in 2022, a markedly steeper trend emerged. Between 2022 and 2024, RO increased by approximately 5.2 research products per year, representing the most rapid growth observed in the decade. Matched applicants experienced a 44.24% increase during this period, and by 2024 reported 32% more RO than their unmatched counterparts (Figures 1 and 2).

## Discussion

The sharp increase in RO following the Step 1 pass/fail transition suggests that research productivity has become a dominant factor in orthopaedic residency selection. With the removal of numerical Step 1 scores, program directors are increasingly relying on variables perceived to distinguish high-achieving applicants, and RO has emerged as a readily quantifiable metric. While engagement in scholarly work is valuable, the magnitude of RO inflation raises questions about whether the current expectations are reasonable or sustainable. Many students now pursue multiple simultaneous research projects, often of limited academic depth, to remain competitive. This shift has implications for medical education, as time diverted to rapid-cycle scholarly production may detract from preclinical foundations and contribute to burnout. The recent decline in USMLE Step 1 pass rates, from 97% in 2020 to 89% in 2025, occurs against this backdrop and may reflect changing student priorities driven by residency selection pressures.^5^

Equity concerns also warrant attention. Applicants from medical schools with limited research infrastructure, without home orthopaedic programs, or without funded mentorship opportunities face structural disadvantages in meeting escalating RO expectations.^6^ These disparities may disproportionately affect students from underrepresented or socioeconomically disadvantaged backgrounds, further narrowing diversity within an already homogenous specialty. Additionally, the validity of RO as a metric is complicated by variability in authorship practices. Prior studies in procedural specialties have documented a rise in total publication counts but a decline in first-author contributions, suggesting a growing prevalence of minimal-contribution or gift authorship.^7^ Similarly, a significant proportion of works listed on ERAS as “submitted” or “in press” may never be published, raising questions about accuracy and verification.^8^ These patterns highlight vulnerabilities in the current system and suggest that RO inflation may not reflect meaningful scholarly development but rather adaptive behavior shaped by systemic pressures.

Recognizing these concerns, the Association of American Medical Colleges (AAMC) has announced forthcoming MyERAS revisions for the 2027 application cycle, including a redesigned Scholarly Work section that allows applicants to identify their three most meaningful works, clearly indicate first-author contributions, and group related outputs.^9^ These reforms aim to reduce publication inflation and improve transparency by shifting emphasis from quantity to substance. However, whether such structural changes will meaningfully alter applicant behavior or program evaluation practices remains unknown.

To address longer-term concerns, several scholars have argued for the integration of holistic review frameworks into residency selection. Kumar et al. propose evaluating applicants through a broader set of domains, including service, leadership, advocacy, and contextual achievements, to recognize excellence not captured by traditional academic metrics.^10^ Applying such frameworks in orthopaedics may mitigate the unintended gatekeeping effects of RO-driven selection and support development of a more diverse, patient-centered workforce. Ultimately, aligning selection practices with the values of the specialty will require balancing scholarly rigor with fairness, transparency, and a commitment to cultivating future surgeons who reflect the patient populations they serve.

## Conclusion

Research output among U.S. orthopaedic residency applicants has risen sharply, with an accelerated increase associated with the Step 1 transition to pass/fail. While scholarly productivity remains important, its expanding role as a screening metric raises ethical and equity concerns and may contribute to applicant burnout and superficial research practices. Continued monitoring of RO trends, thoughtful implementation of ERAS reforms, and incorporation of holistic review principles will be essential to ensuring that orthopaedic residency selection promotes fairness, diversity, and meaningful academic engagement.
